# Two Versus One COVID-19 Vaccine Dose After Prior SARS-CoV-2 Infection: A Nationwide Retrospective Cohort Study

**DOI:** 10.1093/ofid/ofag602

**Published:** 2026-09-22

**Authors:** Hiam Chemaitelly, Houssein H Ayoub, Peter Coyle, Patrick Tang, Mohammad R Hasan, Hadi M Yassine, Asmaa A Al Thani, Zaina Al-Kanaani, Einas Al-Kuwari, Andrew Jeremijenko, Anvar Hassan Kaleeckal, Ali Nizar Latif, Riyazuddin Mohammad Shaik, Hanan F Abdul-Rahim, Gheyath K Nasrallah, Mohamed Ghaith Al-Kuwari, Adeel A Butt, Hamad Eid Al-Romaihi, Mohamed H Al-Thani, Abdullatif Al-Khal, Laith J Abu-Raddad

**Affiliations:** Infectious Disease Epidemiology Group, Weill Cornell Medicine-Qatar, Cornell University, Doha, Qatar; Department of Population Health Sciences, Weill Cornell Medicine, Cornell University, New York, New York, USA; Mathematics Program, Department of Mathematics and Statistics, College of Arts and Sciences, Qatar University, Doha, Qatar; Hamad Medical Corporation, Doha, Qatar; Department of Biomedical Science, College of Health Sciences, QU Health, Qatar University, Doha, Qatar; Wellcome-Wolfson Institute for Experimental Medicine, Queens University, Belfast, UK; Department of Pathology and Laboratory Medicine, University of British Columbia, Vancouver, British Columbia, Canada; Department of Pathology and Molecular Medicine, McMaster University, Hamilton, Ontario, Canada; Department of Biomedical Science, College of Health Sciences, QU Health, Qatar University, Doha, Qatar; Biomedical Research Center, QU Health, Qatar University, Doha, Qatar; Department of Biomedical Science, College of Health Sciences, QU Health, Qatar University, Doha, Qatar; Biomedical Research Center, QU Health, Qatar University, Doha, Qatar; Hamad Medical Corporation, Doha, Qatar; Hamad Medical Corporation, Doha, Qatar; Hamad Medical Corporation, Doha, Qatar; Hamad Medical Corporation, Doha, Qatar; Hamad Medical Corporation, Doha, Qatar; Hamad Medical Corporation, Doha, Qatar; Department of Public Health, College of Health Sciences, QU Health, Qatar University, Doha, Qatar; Department of Biomedical Science, College of Health Sciences, QU Health, Qatar University, Doha, Qatar; Biomedical Research Center, QU Health, Qatar University, Doha, Qatar; Primary Health Care Corporation, Doha, Qatar; College of Medicine, Qatar University, Doha, Qatar; Department of Population Health Sciences, Weill Cornell Medicine, Cornell University, New York, New York, USA; Hamad Medical Corporation, Doha, Qatar; Department of Medicine, Weill Cornell Medicine, Cornell University, New York, New York, USA; Ministry of Public Health, Doha, Qatar; Ministry of Public Health, Doha, Qatar; Hamad Medical Corporation, Doha, Qatar; Infectious Disease Epidemiology Group, Weill Cornell Medicine-Qatar, Cornell University, Doha, Qatar; Department of Population Health Sciences, Weill Cornell Medicine, Cornell University, New York, New York, USA; Biomedical Research Center, QU Health, Qatar University, Doha, Qatar; College of Health and Life Sciences, Hamad bin Khalifa University, Doha, Qatar

**Keywords:** BNT162b2, immunity, mRNA-1273, omicron, reinfection

## Abstract

**Background:**

Immune protection against severe acute respiratory syndrome coronavirus 2 (SARS-CoV-2) varies by prior infection and vaccination history. This study evaluated whether 2 coronavirus disease (COVID-19) vaccine doses after prior infection provide additional protection compared with a single dose.

**Methods:**

A national matched retrospective cohort study was conducted in Qatar from December 1, 2020-March 3, 2025. Incidence of SARS-CoV-2 reinfection and severe COVID-19 at reinfection was compared between 61 854 previously infected individuals who completed primary-series vaccination (2-dose cohort) and 61 854 matched individuals who received a single dose (single-dose cohort).

**Results:**

After 360 days of follow-up, cumulative incidence of reinfection was 5.0% (95% CI, 3.8%–6.7%) in the 2-dose cohort and 7.7% (95% CI, 6.1%–9.6%) in the single-dose cohort. No reinfections progressed to severe, critical, or fatal COVID-19 in either cohort. Relative vaccine effectiveness (rVE) of 2 doses vs 1 dose was 38.9% (95% CI, 20.4%–53.2%). The rVE was 26.9% (95% CI, −12.7%–53.4%) within <24 days, 51.8% (95% CI, 19.2%–71.2%) during Days 24–179, and 36.8% (95% CI, 0.9%–59.7%) at ≥180 days of follow-up. Subgroup analyses by time between prior infection and vaccination, prior infection variant, and vaccine type yielded broadly similar rVE estimates, while analyses stratified by age and clinical vulnerability suggested higher point estimates among older and clinically vulnerable individuals.

**Conclusions:**

Among individuals with prior infection, completion of primary-series vaccination provided modest additional protection against reinfection compared with a single dose. These findings support risk-based dose-optimization strategies in settings with constrained vaccine supply.

## Teaser

This study found that among previously infected individuals, completion of 2-dose primary-series vaccination conferred modest additional protection against reinfection compared with a single dose, supporting risk-based, dose-optimization strategies in settings with constrained vaccine supply.

Different immune histories arising from vaccination, natural infection, or their combination can confer heterogeneous and time-varying protection against infection and severe disease [1–7]. Yet, robust quantification of these effects is frequently constrained by limited resources and by the methodological challenges of disentangling overlapping and evolving sources of immunity [4, 8, 9]. During public health emergencies, such as the coronavirus disease (COVID-19) pandemic, and in settings with constrained vaccine supply, policy decisions may favor strategies that maximize equitable population-level coverage of partial protection rather than individual-level immune optimization [10–12]. In the absence of definitive evidence on the magnitude and durability of protection across immune states, such decisions carry an inherent risk of suboptimal policy making.

Although COVID-19 mRNA vaccines were authorized as a 2-dose primary series [13–15], early supply limitations and the urgency to rapidly protect large high-risk populations prompted some countries to recommend a single vaccine dose for individuals with documented prior severe acute respiratory syndrome coronavirus 2 (SARS-CoV-2) infection, effectively treating prior infection as immunologically equivalent to 1 vaccine dose [16, 17]. This pragmatic policy response underscores the need for empirical evidence on whether completing the primary series confers meaningful incremental protection among previously infected individuals.

Qatar offers a well-suited setting to address this evidence gap through a retrospective analysis of immune histories in its vaccinated population, leveraging comprehensive, linked national SARS-CoV-2 databases that enable population-level characterization of immune states and robust ascertainment of clinical outcomes [6, 18]. This setting is also informative because the national vaccination program retained the standard 2-dose primary-series schedule for mRNA vaccines regardless of prior infection status [19, 20], allowing comparison of 1 versus 2 postinfection vaccine doses within the same population.

Using a nationwide cohort design, this study assessed whether receipt of 2 COVID-19 vaccine doses following natural infection provided additional protection against SARS-CoV-2 reinfection and severe COVID-19 compared with a single dose among individuals with 1 documented prior infection, thereby directly contrasting 2 scientifically and policy-relevant immune histories.

## METHODS

### Study Population and Data Sources

This study was conducted in the population of Qatar from December 1, 2020, at the start of the national COVID-19 vaccination campaign [21], to March 3, 2025. Data were obtained from Qatar's integrated national digital health platform (Supplementary Section 1), which captures SARS-CoV-2–related records nationwide, including laboratory testing, vaccination, hospitalizations, and deaths. The platform includes all polymerase chain reaction (PCR) test results and, from January 5, 2022, onward, all medically supervised rapid antigen test results, irrespective of testing location or healthcare facility, ensuring comprehensive capture of SARS-CoV-2–related healthcare encounters (Supplementary Section 2).

Until October 31, 2022, Qatar implemented extensive SARS-CoV-2 testing, with approximately 5% of the population tested weekly, largely for routine screening and travel-related purposes [19, 22]. From November 1, 2022, onward, testing intensity declined to below 1% of the population per week [23]. Nevertheless, throughout the study period, most SARS-CoV-2 infections in Qatar were identified through routine rather than symptom-driven testing (Supplementary Section 1) [19, 22].

COVID-19 vaccination in Qatar was provided free of charge, regardless of citizenship or residency status, with initial prioritization of older adults and individuals with coexisting conditions (Supplementary Section 1) [19, 20]. All vaccinations were recorded nationally and linked within the digital health platform. The platform also contains data on coexisting conditions for individuals accessing care through the universal public healthcare system since its establishment in 2013 (Supplementary Section 3).

Demographic information, including sex, age, and nationality, was extracted from the national health registry. Qatar's population is highly diverse, with expatriates comprising approximately 89% of residents and representing more than 150 nationalities [24]. Detailed descriptions of the study population and data sources have been published elsewhere [6, 7, 19, 22, 24–26].

### Study Design

A national, matched, retrospective cohort study was conducted to compare the incidence of SARS-CoV-2 reinfection and of severe COVID-19 among individuals with a single documented prior infection who received a 2-dose COVID-19 primary-series vaccination (2-dose cohort) with that among individuals who received a single vaccine dose after infection (single-dose cohort). The study design builds on previously developed and implemented cohort study methodologies in Qatar's population [4, 7, 19, 20, 22–25, 27–35].

Incidence of SARS-CoV-2 reinfection was defined as any PCR-positive or rapid-antigen-positive test occurring after the start of follow-up, irrespective of symptomatic presentation. Infection severity was classified according to the World Health Organization criteria as severe [36], critical [36], or fatal [37] COVID-19, based on independent chart reviews conducted by trained medical personnel [38]. As per national protocol, SARS-CoV-2–positive hospital admissions underwent standardized severity assessments every 3 days until discharge or death [38, 39]. Each case was assigned its highest level of severity, prioritized as fatal, critical, and then severe disease [38, 39].

### Cohorts' Eligibility and Matching

Individuals were eligible for inclusion in the 2-dose cohort if they received 2 COVID-19 vaccine doses after a prior SARS-CoV-2 infection, and in the single-dose cohort if they received 1 vaccine dose after a prior infection. Prior infection was defined as a SARS-CoV-2–positive test occurring ≥90 days before receipt of the second vaccine dose in the 2-dose cohort or ≥90 days before receipt of the first vaccine dose in the single-dose cohort. This 90-day interval was used to minimize misclassification of prolonged viral shedding from a previous infection as an incident infection [40–42]. Prior infections were classified as pre-omicron if they occurred before 19 December 2021, marking the onset of the first omicron wave in Qatar [22], and as omicron thereafter.

The study population was restricted to individuals with 1 documented SARS-CoV-2 infection prior to vaccination to reduce heterogeneity in infection-induced immunity, which could otherwise obscure the incremental effect of an additional vaccine dose. Individuals who received the pediatric 10 µg BNT162b2 formulation, mixed vaccine types across doses, or had an unascertained date of death were excluded. Eligibility criteria were applied independently within each cohort according to the relevant index vaccination date. Accordingly, the eligible cohort counts were not expected to be nested.

Cohorts were matched exactly 1-to-1 by sex, 10-year age group, nationality, number of coexisting conditions (categorized as 0 to ≥6), prior infection variant (pre-omicron vs omicron), time between prior infection and vaccination (<6 months, 6–11 months, and ≥12 months at the time of second-dose vaccination for individuals in the 2-dose cohort and first-dose vaccination for individuals in the single-dose cohort), and vaccine type (BNT162b2, mRNA-1273, AZD1222, or other). In addition, individuals in the 2-dose cohort who received their second vaccine dose in a given calendar week were matched to individuals in the single-dose cohort who received their first vaccine dose in the same calendar week. This design aligned the timing of the most recent immunological event initiating follow-up, thereby enforcing comparability between cohorts.

Iterative matching was implemented to ensure that, at the start of follow-up, individuals were alive, had maintained their vaccination status, and had no documented SARS-CoV-2 infection within the preceding 90 days. The matching strategy was designed to balance observed confounders that could influence infection risk across exposure groups [24, 43–45], with matching factors informed by earlier studies conducted in Qatar's population [19, 20, 31]. Matching was performed using the Stata *ccmatch* command, supplemented with eligibility constraints, and iterated until no additional eligible matched pairs could be identified.

Under this design, individuals in the single-dose cohort contributed follow-up time until receipt of a second vaccine dose, after which they could, if rematched, contribute additional follow-up time to the 2-dose cohort upon completion of the primary vaccination series.

### Cohorts' Follow-Up

Follow-up began on the calendar date of receipt of the second vaccine dose for individuals in the 2-dose cohort. To preserve exchangeability [25, 46], both members of each matched pair were censored at the earliest receipt of an additional vaccine dose.

Individuals were followed until the first occurrence of any of the following events: a documented SARS-CoV-2 reinfection (irrespective of symptoms), third-dose vaccination for individuals in the 2-dose cohort (with matched-pair censoring), second-dose vaccination for individuals in the single-dose cohort (with matched-pair censoring), death, or administrative end of follow-up at the end of the study.

### Oversight

The Institutional Review Boards at Hamad Medical Corporation and Weill Cornell Medicine–Qatar approved this retrospective study with a waiver of informed consent. The study was reported according to the Strengthening the Reporting of Observational Studies in Epidemiology (Supplementary Table 1).

### Statistical Analysis

Eligible and matched cohorts were summarized using frequency distributions and measures of central tendency and compared using standardized mean differences (SMDs). An SMD of ≤0.1 indicated adequate matching [47]. The cumulative incidence of reinfection, defined as the proportion of individuals at risk whose primary endpoint during follow-up was a SARS-CoV-2 reinfection, was estimated using the Kaplan–Meier estimator method [48].

The incidence rate of reinfection for each cohort, defined as the number of incident reinfections divided by the number of person-weeks contributed by all individuals in the cohort, was estimated along with the corresponding 95% confidence interval (CI), using a Poisson log-likelihood regression model with the Stata *stptime* command.

The overall adjusted hazard ratio (aHR) comparing the incidence of SARS-CoV-2 reinfection between cohorts, with corresponding 95% CIs, was estimated using a Cox regression model adjusted for the matching factors and implemented with the Stata *stcox* command. This adjustment was applied to ensure precise and unbiased estimation of standard errors and variance [49]. An additional adjustment for testing rate was included to account for potential differences in infection detection arising from variations in routine testing practices between cohorts. Confidence intervals were not adjusted for multiplicity. E-values were calculated for the overall aHR to assess the minimum strength of association that an unmeasured confounder would need to have with both vaccination status and reinfection, beyond the measured covariates included in the model, to fully explain the observed association.

The proportional hazards assumption was assessed using Schoenfeld residuals and log–log survival plots and was satisfied. Nevertheless, because the overall aHR represents a weighted average of potentially time-varying hazard ratios [50], additional analyses were conducted to examine temporal variation in reinfection risk. Specifically, aHRs were estimated for predefined follow-up intervals (<24 days, days 24–179, and ≥180 days) using separate Cox regression models, with failures restricted to corresponding time intervals. The <24-day interval was chosen because the median follow-up time was 23 days.

Relative vaccine effectiveness (rVE) against reinfection and associated 95% CIs were derived from the aHRs as 1–aHR when the aHR was <1, and as (1/aHR)–1 when the aHR was ≥1 [7, 51]. This transformation yields a symmetric scale ranging from −100% to 100% for both negative and positive effectiveness, allowing meaningful interpretation of both protective and adverse associations.

Subgroup analyses estimated the overall aHR stratified by time between prior infection and vaccination, prior infection variant, vaccine type, age group (<50 years vs ≥50 years), and clinical vulnerability status. Individuals were classified as less clinically vulnerable to severe COVID-19 if they were <50 years of age and had 1 or no coexisting conditions, and as more clinically vulnerable to severe COVID-19 if they were either ≥50 years of age or <50 years of age but with ≥2 coexisting conditions [7, 35].

Sensitivity analyses were conducted to assess the robustness of the findings. First, Cox regression models were fitted with interaction terms between vaccination cohort and either follow-up interval or selected subgroup variables, as applicable, to assess whether the hazard ratio comparing 2 versus 1 vaccine dose varied across strata. Stratum-specific aHRs were derived from these models, and *P* values for interaction were obtained using Wald tests of the interaction terms, with joint tests applied when the interacting variable had more than 2 levels. Second, the main analysis and the analysis stratified by time since vaccination were refitted using robust standard errors clustered at the individual level to account for within-person correlation among individuals contributing to both matched cohorts.

Statistical analyses were performed using Stata/SE version 19.0 (Stata Corporation, College Station, TX, USA).

## RESULTS

### Study Population

Supplementary Figure 1 illustrates the study population selection process. Table 1 summarizes the baseline characteristics of the eligible and matched cohorts. As the study was conducted in Qatar's national population, the study population reflects the country's internationally diverse and predominantly male demographic profile.

**Table 1.** Baseline Characteristics of Eligible and Matched Cohorts in the Study Comparing Individuals With Prior SARS-CoV-2 Infection Who Received Two Vaccine Doses With Those Who Received a Single Dose

Characteristics	Eligible Cohorts^a^			Matched Cohorts^b^		
	Two-Dose	Single-Dose	SMD^c^	Two-Dose	Single-Dose	SMD^c^
	N = 211 805	N = 193 435		N = 61 854	N = 61 854	
Median age (IQR)—years	38 (32–46)	38 (32–46)	0.00^d^	37 (31–44)	37 (31–44)	0.02^d^
Age group—no. (%)						
0–19 years	9453 (4.5)	8642 (4.5)	0.01	2925 (4.7)	2925 (4.7)	0.00
20–29 years	28 033 (13.2)	26 299 (13.6)		8630 (14.0)	8630 (14.0)	
30–39 years	77 572 (36.6)	70 452 (36.4)		25 137 (40.6)	25 137 (40.6)	
40–49 years	59 683 (28.2)	53 915 (27.9)		16 972 (27.4)	16 972 (27.4)	
50–59 years	26 471 (12.5)	24 278 (12.6)		6328 (10.2)	6328 (10.2)	
60−69 years	8410 (4.0)	7801 (4.0)		1606 (2.6)	1606 (2.6)	
70+ years	2183 (1.0)	2048 (1.1)		256 (0.4)	256 (0.4)	
Sex						
Male	157 879 (74.5)	145 000 (75.0)	0.01	47 204 (76.3)	47 204 (76.3)	0.00
Female	53 926 (25.5)	48 435 (25.0)		14 650 (23.7)	14 650 (23.7)	
Nationality^e^						
Bangladeshi	23 682 (11.2)	21 851 (11.3)	0.03	7374 (11.9)	7374 (11.9)	0.00
Egyptian	10 102 (4.8)	9185 (4.7)		2684 (4.3)	2684 (4.3)	
Filipino	20 706 (9.8)	17 849 (9.2)		6465 (10.5)	6465 (10.5)	
Indian	55 753 (26.3)	50 982 (26.4)		18 391 (29.7)	18 391 (29.7)	
Nepalese	27 394 (12.9)	25 962 (13.4)		8068 (13.0)	8068 (13.0)	
Pakistani	10 904 (5.1)	10 176 (5.3)		3120 (5.0)	3120 (5.0)	
Qatari	20 683 (9.8)	19 246 (9.9)		8176 (13.2)	8176 (13.2)	
Sri Lankan	7706 (3.6)	6887 (3.6)		1991 (3.2)	1991 (3.2)	
Sudanese	5536 (2.6)	5041 (2.6)		1384 (2.2)	1384 (2.2)	
Other nationalities^f^	29 339 (13.9)	26 256 (13.6)		4201 (6.8)	4201 (6.8)	
Coexisting conditions						
0	168 185 (79.4)	153 834 (79.5)	0.01	52 846 (85.4)	52 846 (85.4)	0.00
1	22 863 (10.8)	20 794 (10.7)		5438 (8.8)	5438 (8.8)	
2	10 823 (5.1)	9686 (5.0)		2125 (3.4)	2125 (3.4)	
3	4595 (2.2)	4194 (2.2)		693 (1.1)	693 (1.1)	
4	2495 (1.2)	2277 (1.2)		354 (0.6)	354 (0.6)	
5	1454 (0.7)	1329 (0.7)		178 (0.3)	178 (0.3)	
≥6	1390 (0.7)	1321 (0.7)		220 (0.4)	220 (0.4)	
Vaccine type						
BNT162b2	121 585 (57.4)	112 216 (58.0)	0.06	39 545 (63.9)	39 545 (63.9)	0.00
mRNA-1273	86 892 (41.0)	76 975 (39.8)		21 903 (35.4)	21 903 (35.4)	
AZD1222	3240 (1.5)	3861 (2.0)		396 (0.6)	396 (0.6)	
Other vaccine types^g^	88 (0.04)	383 (0.2)		10 (0.02)	10 (0.02)	
Prior infection variant						
Pre-omicron	209 708 (99.0)	189 261 (97.8)	0.09	61 192 (98.9)	61 192 (98.9)	0.00
Omicron	2097 (1.0)	4174 (2.2)		662 (1.1)	662 (1.1)	
Time between prior infection and vaccination^h^						
<6 months	70 499 (33.3)	58 775 (30.4)	0.17	20 109 (32.5)	20 109 (32.5)	0.00
6–11 months	91 760 (43.3)	99 421 (51.4)		28 647 (46.3)	28 647 (46.3)	
≥12 months	49 546 (23.4)	35 239 (18.2)		13 098 (21.2)	13 098 (21.2)	

Abbreviations: IQR, interquartile range; SARS-CoV-2, severe acute respiratory syndrome coronavirus 2; SMD, standardized mean difference.

^a^Eligible 2-dose and single-dose cohorts were defined independently, each according to its relevant index vaccination date: Dose 2 for the 2-dose cohort and dose 1 for the single-dose cohort (Supplementary Figure 1). Accordingly, the eligible cohort counts are not expected to be nested.

^b^Persons in the 2-dose cohort were matched exactly 1-to-1 to persons in the single-dose cohort by sex, 10-year age group, nationality, number of coexisting conditions, prior infection variant, time between prior infection and vaccination, vaccine type, and calendar week of vaccination (calendar week of the second dose in the 2-dose cohort matched to the calendar week of the first dose in the single-dose cohort). Each matched pair was followed from the date of second-dose vaccination for the individual in the 2-dose cohort.

^c^SMD is the difference in the mean of a covariate between groups divided by the pooled standard deviation. An SMD of ≤0.1 indicates adequate matching.

^d^SMD is for the mean difference between groups divided by the pooled standard deviation.

^e^Nationalities were chosen to represent the most populous groups in Qatar.

^f^These comprise up to 134 and 50 other nationalities in the unmatched and matched analyses, respectively.

^g^These include mRNA-1273.214 (Spikevax bivalent original/omicron BA.1), mRNA-1273.222 (Spikevax bivalent original/omicron BA.4/BA.5), BNT162b2 original/omicron BA.4/BA.5 (Comirnaty original/omicron BA.4/BA.5), and mRNA-1273.815 (Spikevax XBB.1.5).

^h^Vaccination refers to the second dose in the 2-dose cohort and the first dose in the single-dose cohort.

Between December 1, 2020, and March 3, 2025, a total of 2 384 698 individuals received at least 1 COVID-19 vaccine dose, of whom 2 313 982 also received a second dose. The median dates of administration were May 17, 2021, for the first dose and June 9, 2021, for the second dose. The median interval between the first and second doses was 28 days (interquartile range [IQR], 21–28 days), being 21 days (IQR, 21–22 days) for BNT162b2 and 28 days (IQR, 28–30 days) for mRNA-1273.

Each matched cohort comprised 61 854 individuals, with 19 157 individuals (31.0%) contributing to both matched cohorts at different vaccination states. The median vaccination date was June 9, 2021, for the first-dose vaccination in the single-dose cohort and June 11, 2021, for the second-dose vaccination in the 2-dose cohort. The median duration of follow-up was 23 days (IQR, 20–28 days) in both cohorts (Figure 1). The median interval between the first and second doses in the 2-dose cohort was 22 days (IQR, 21–28 days), being 21 days (IQR, 21–22 days) for BNT162b2 and 28 days (IQR, 28–35 days) for mRNA-1273.

**Figure 1.**
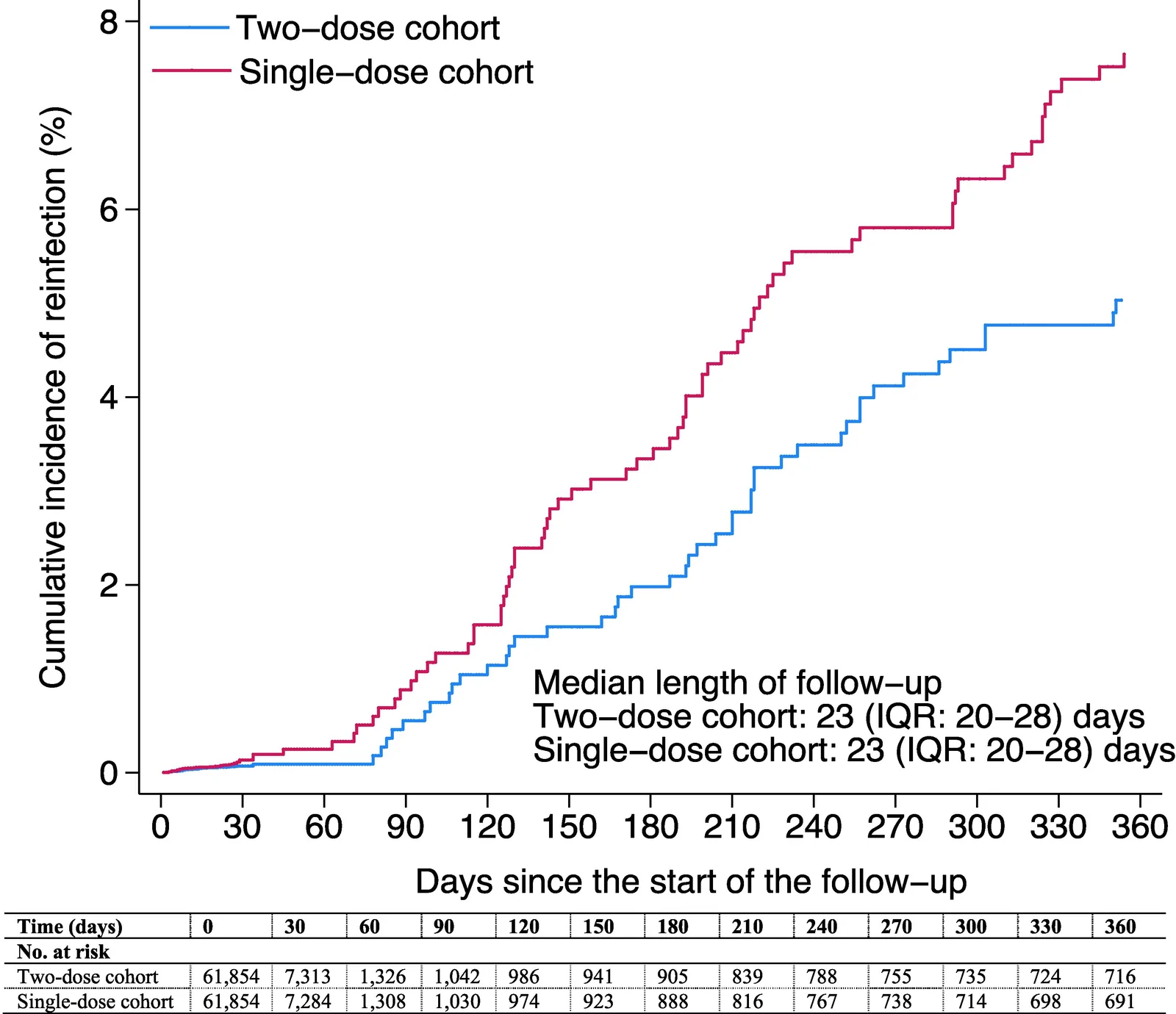
Cumulative incidence of SARS-CoV-2 reinfection comparing individuals with prior SARS-CoV-2 infection who received 2 vaccine doses with those who received a single dose. Abbreviations: IQR, interquartile range; SARS-CoV-2, severe acute respiratory syndrome coronavirus 2.

Of the 61 854 matched single-dose cohort participants, 54 306 (87.8%) had a documented second dose within 30 days, 6677 (10.8%) had a documented second dose 31 or more days after the first dose, and 871 (1.4%) had no documented second dose during the study period. Supplementary Table 2 presents the characteristics of individuals in the matched single-dose cohort according to subsequent receipt and timing of a documented second vaccine dose.

### Incidence of SARS-CoV-2 Reinfection

During follow-up, 93 SARS-CoV-2 reinfections were documented in the 2-dose cohort compared with 139 in the single-dose cohort (Table 2A; Supplementary Figure 1). None of the reinfections progressed to severe, critical, or fatal COVID-19. The cumulative incidence of reinfection was 5.0% (95% CI, 3.8%–6.7%) in the 2-dose cohort and 7.7% (95% CI, 6.1%–9.6%) in the single-dose cohort after 360 days of follow-up (Figure 1).

**Table 2.** Hazard Ratios and Relative Vaccine Effectiveness for SARS-CoV-2 Reinfection Comparing 2 Versus 1 Vaccine Dose Among Individuals With Prior SARS-CoV-2 Infection A) in Main Analysis and B) Stratified by Time Since Vaccination

Epidemiological Measure	Two-Dose Cohort^a^	Single-Dose Cohort^a^
A) Main analysis		
Sample size	61 854	61 854
Total follow-up time (person-weeks)	326 111	322 142
Incident infections	93	139
Incidence rate of reinfection (per 10 000 person-weeks; 95% CI)	2.9 (2.3–3.5)	4.3 (3.7–5.1)
Unadjusted hazard ratio for SARS-CoV-2 reinfection (95% CI)	0.66 (0.51–0.86)	
Adjusted hazard ratio for SARS-CoV-2 reinfection (95% CI)^b^	0.61 (0.47–0.80)	
Relative vaccine effectiveness against SARS-CoV-2 reinfection in % (95% CI)	38.9 (20.4–53.2)	
B) Time since vaccination^c^		
<24 days		
Adjusted hazard ratio for SARS-CoV-2 reinfection (95% CI)^b^	0.73 (0.47–1.15)	
Relative vaccine effectiveness against SARS-CoV-2 reinfection in % (95% CI)	26.9 (−12.7–53.4)	
24–179 days		
Adjusted hazard ratio for SARS-CoV-2 reinfection (95% CI)^b^	0.48 (0.29–0.81)	
Relative vaccine effectiveness against SARS-CoV-2 reinfection in % (95% CI)	51.8 (19.2–71.2)	
≥180 days		
Adjusted hazard ratio for SARS-CoV-2 reinfection (95% CI)^b^	0.63 (0.40–0.99)	
Relative vaccine effectiveness against SARS-CoV-2 reinfection in % (95% CI)	36.8 (0.9–59.7)	

Abbreviations: CI, confidence interval; SARS-CoV-2, severe acute respiratory syndrome coronavirus 2.

^a^Persons in the 2-dose cohort were matched exactly 1-to-1 to persons in the single-dose cohort by sex, 10-year age group, nationality, number of coexisting conditions, prior infection variant, time between infection and vaccination, vaccine type, and calendar week of vaccination (calendar week of the second dose in the 2-dose cohort matched to the calendar week of the first dose in the single-dose cohort). Each matched pair was followed from the date of second-dose vaccination for the individual in the two-dose cohort.

^b^Adjusted for sex, 10-year age group, nationality, number of coexisting conditions, prior infection variant, time between prior infection and vaccination, vaccine type, and the calendar week of vaccination (calendar week of the second dose in the 2-dose cohort matched to the calendar week of the first dose in the single-dose cohort), and testing rate, as applicable depending on strata.

^c^Vaccination refers to the second dose in the 2-dose cohort and the first dose in the single-dose cohort.

The overall aHR comparing the incidence of SARS-CoV-2 reinfection in the 2-dose cohort with that in the single-dose cohort was 0.61 (95% CI, 0.47–0.80), after adjustment for sex, 10-year age group, nationality, number of coexisting conditions, prior infection variant, time between prior infection and vaccination, vaccine type, calendar week of vaccination, and testing rate (Table 2A). This corresponded to an rVE of 38.9% (95% CI, 20.4%–53.2%).

In the quantitative bias analysis, the E-value for the overall aHR was 2.66 for the point estimate and 1.81 for the confidence limit closest to the null, indicating that an unmeasured confounder would need to be associated with both vaccination status and reinfection by at least 2.66-fold each, beyond the measured covariates, to fully explain the observed association.

In analyses stratified by time since vaccination, the aHR was 0.73 (95% CI, 0.47–1.15) within <24 days, 0.48 (95% CI, 0.29–0.81) 24-179 days, and 0.63 (95% CI, 0.40–0.99) at ≥180 days after the start of follow-up (Table 2B). Corresponding rVE estimates were 26.9% (95% CI, –12.7%–53.4%), 51.8% (95% CI, 19.2%–71.2%), and 36.8% (95% CI, 0.9%–59.7%), respectively.

### Subgroup and Sensitivity Analyses

Subgroup analyses stratified by time between prior infection and vaccination, prior infection variant, and vaccine type yielded broadly similar rVE estimates across strata, although precision was limited by wide CIs (Figure 2; Supplementary Table 3). Analyses stratified by age group and clinical vulnerability suggested higher point estimates among older and more clinically vulnerable individuals; however, these estimates were similarly limited by wide CIs (Figure 3; Supplementary Table 4).

**Figure 2.**
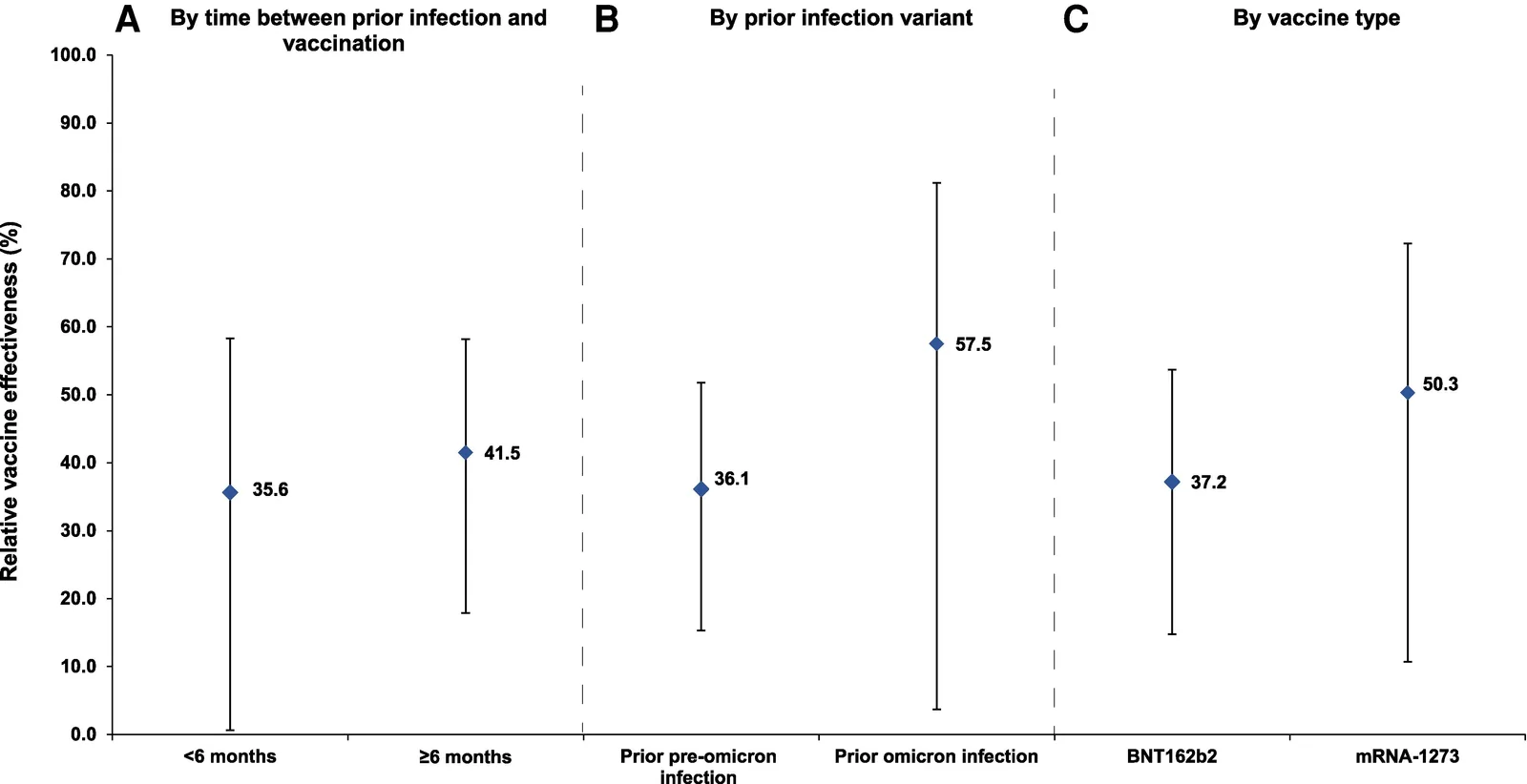
Subgroup analysis. Relative vaccine effectiveness against reinfection comparing 2 versus 1 vaccine dose among individuals with prior SARS-CoV-2 infection, stratified by (*A*) time between prior infection and vaccination, (*B*) prior infection variant, and (*C*) vaccine type. Error bars denote 95% confidence intervals. Abbreviation: SARS-CoV-2, severe acute respiratory syndrome coronavirus 2.

**Figure 3.**
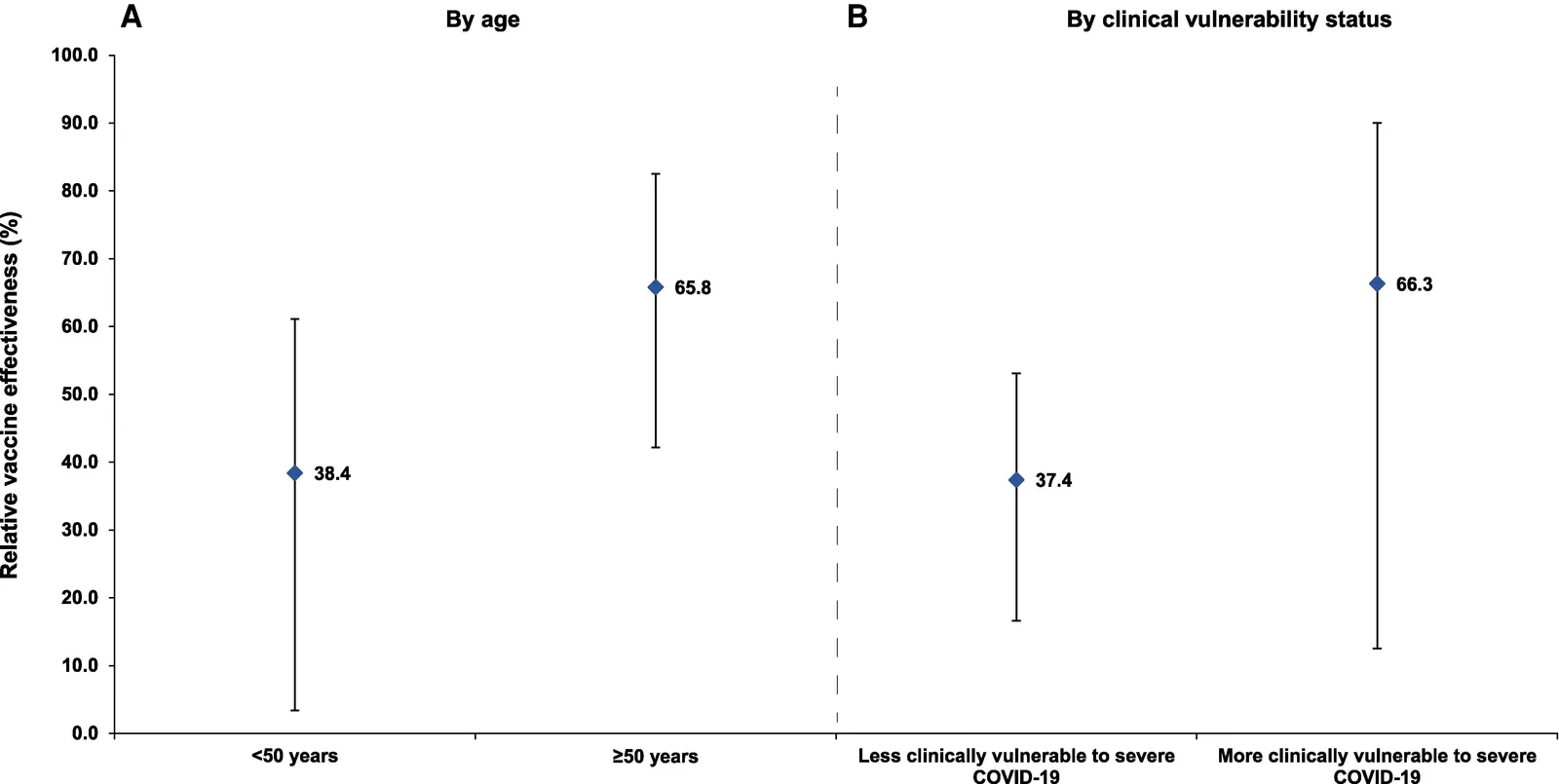
Subgroup analysis. Relative vaccine effectiveness against reinfection comparing individuals with prior SARS-CoV-2 infection who received 2 vaccine doses with those who received a single dose, stratified by (*A*) age and (*B*) clinical vulnerability status. Error bars denote 95% confidence intervals. Abbreviations: COVID-19, coronavirus disease; SARS-CoV-2, severe acute respiratory syndrome coronavirus 2.

Sensitivity analyses were consistent with the main findings. In interaction-model sensitivity analyses, rVE estimates were broadly consistent with the main, time-stratified, and subgroup analyses. There was no evidence that the hazard ratio comparing 2 vs 1 vaccine dose varied significantly by follow-up interval, time between prior infection and vaccination, prior infection variant, vaccine type, age, or clinical vulnerability status (Supplementary Table 5). Therefore, the overall estimate remains a reasonable summary of the association, and the observed differences in point estimates across strata should be interpreted cautiously as exploratory findings.

Results were also materially similar when the main analysis and the analysis stratified by time since vaccination were refitted using robust standard errors clustered at the individual level, with only slightly wider 95% CIs (Supplementary Table 6).

## DISCUSSION

Completion of a 2-dose COVID-19 primary vaccination series among previously infected individuals was associated with an approximately 40% relative vaccine effectiveness against reinfection compared with receipt of a single vaccine dose. No reinfections progressed to severe, critical, or fatal COVID-19 in either cohort, a reassuring finding consistent with existing evidence that natural infection alone confers strong and durable protection against severe reinfection [6, 29, 52, 53]. The addition of a single vaccine dose to infection-induced immunity may further achieve a high level of protection against severe disease [22, 54], potentially leaving a limited scope for a second dose to provide further measurable benefit.

The added protection against reinfection conferred by the second dose was modest during the first 3 weeks following vaccination (Figure 1), plausibly reflecting already high neutralizing antibody titers generated by the combination of natural infection and a single vaccine dose [55, 56]. During this early period, the second dose may have provided little short-term incremental benefit. Over subsequent follow-up, however, protection diverged, with the two-dose series offering greater protection against reinfection than a single dose (Figure 1), persisting for up to 1 year of follow-up. This pattern suggests that, among previously infected individuals, the principal contribution of the second dose may be to enhance the durability rather than the peak magnitude of protection.

These findings differ from observations in populations without restriction by prior infection history, in which protection following a single dose was initially lower and accrued more gradually than after completion of the 2-dose series [57], but converged toward similar levels within a few months [57]. The contrasting pattern observed here supports the hypothesis that prior infection reshapes the immune landscape at the time of vaccination, resulting in a distinct immune response and trajectory of protection following subsequent doses.

The incremental protection associated with a second dose appeared consistent across strata defined by time between prior infection and vaccination, prior infection variant, and vaccine type. Point estimates were higher among older adults and clinically vulnerable individuals, although tests for interaction did not provide evidence that the effect differed significantly by age or clinical vulnerability status. These findings should therefore be interpreted cautiously, given the limited number of events and wide CIs in these strata. Nonetheless, greater marginal benefit in older or clinically vulnerable populations remains biologically plausible, as immunosenescence and coexisting conditions can attenuate both the magnitude and durability of immune responses to infection and vaccination [58–60].

From a policy perspective, these results support targeted, risk-based dose-optimization strategies. They provide empirical justification for policies adopted by some countries during periods of COVID-19 vaccine scarcity that prioritized first-dose coverage and deferred second doses among individuals with prior infection [16, 17]. In settings with constrained vaccine supply or competing public health priorities, routine administration of multiple doses to previously infected individuals may yield limited additional benefit for preventing severe disease. Instead, prioritizing completion of the primary series among older adults and clinically vulnerable populations remains a reasonable strategy to support population-level impact while conserving resources. Nonetheless, the reduction in reinfection risk associated with a second dose may remain relevant in specific contexts, such as protecting essential workers, alleviating healthcare system strain, or reducing workforce disruption during epidemic surges.

This study has limitations. Despite the relatively large cohort sizes, the vast majority of individuals in the single-dose cohort received a second vaccine dose within 1 month, in line with mRNA vaccine schedules [13, 14]. This rapid progression substantially shortened follow-up time in both cohorts, limiting statistical power for subgroup analyses, resulting in reduced precision and wide CIs, and preventing evaluation of protection beyond 1 year of follow-up. Consequently, longer-term patterns remain uncharacterized, including whether protection associated with the 2 immune histories may eventually converge.

This limited follow-up also means that the 360-day cumulative incidence estimates should be interpreted cautiously, as few individuals remained at risk at later follow-up times, making these estimates less precise and more susceptible to selection bias due to risk-set depletion. This rapid depletion of the single-dose cohort may have introduced selection bias if individuals who remained in the single-dose state differed systematically from those who completed the primary series on schedule. However, nearly all participants in the single-dose cohort eventually received a documented second dose, although at a later time, with only 1.4% having no documented second dose during the study period.

The matching strategy was designed to address key measured confounding pathways between vaccination status and reinfection risk. Sex, age group, and nationality were used to balance demographic, socioeconomic, and behavioral risk differences [24, 61]; number of coexisting conditions was used to balance clinical vulnerability and potential vaccine-prioritization differences; prior infection variant and time between prior infection and vaccination were used to balance differences in infection-induced immunity; vaccine type was used to account for differences by vaccine product; and calendar week of vaccination was used to balance calendar-time effects, including epidemic phase, infection pressure, circulating variants, testing practices, and public health measures. However, as with all observational studies, confounding from unmeasured factors cannot be excluded [62].

Data limitations precluded matching on factors such as occupation, geographic location, or individual behaviors that may influence infection risk [63]. Differential postvaccination behavior, such as potentially greater risk compensation after dose 1 than after dose 2, could have increased exposure risk during single-dose follow-up, thereby overestimating the incremental protection associated with the second dose. Because the study was conducted within a specific national population, generalizability to settings with different demographic structures and epidemiologic contexts is uncertain.

Nevertheless, several features of the setting and study design may have mitigated concerns about residual bias and baseline exposure-risk differences. Qatar's highly urbanized city-state structure may have minimized geographic heterogeneity, and nationality, age, and sex—key matching variables—act as strong proxies for socioeconomic status in this setting, potentially capturing some of the unmeasured socioeconomic determinants of exposure [24, 43–45, 64]. Importantly, the matching approach used here has been validated in prior studies employing negative control outcomes in the same population and across multiple epidemiologic designs [18–20, 65, 66], supporting its reliability in balancing exposure risk and producing robust vaccine effectiveness estimates.

This study has strengths. It leveraged comprehensive, linked national health databases capturing SARS-CoV-2 testing, vaccination, hospitalizations, and deaths, thereby minimizing selection bias and outcome misclassification. The nationwide scope, encompassing a demographically diverse population, together with exact matching on key demographic, clinical, temporal, and immunologic factors, ensured close comparability between cohorts and strengthened internal validity. The study design also allowed individuals to cross over and contribute follow-up time to both exposure groups according to vaccination status, improving statistical efficiency while preserving validity through matched-pair censoring. Finally, the analyses relied on national databases that have been extensively validated and used in prior vaccine effectiveness studies, supporting the robustness and reliability of the findings across outcomes.

## CONCLUSION

Among individuals with prior SARS-CoV-2 infection, completion of a 2-dose COVID-19 primary vaccination series conferred modest additional protection against reinfection compared with receipt of a single dose. No reinfections progressed to severe, critical, or fatal COVID-19 in either cohort. These findings indicate that, in the presence of strong infection-induced immunity, a single vaccine dose may be sufficient to achieve a high level of protection against severe outcomes, while a second dose primarily enhances the durability of protection against reinfection. The results support risk-based and context-specific dose-optimization strategies in settings with constrained vaccine supply, with prioritization of additional doses for older adults and clinically vulnerable populations most likely to derive meaningful benefit.

## Supplementary Material

ofag602_Supplementary_Data

## Supplementary Data

Supplementary materials are available at *Open Forum Infectious Diseases* online. Consisting of data provided by the authors to benefit the reader, the posted materials are not copyedited and are the sole responsibility of the authors, so questions or comments should be addressed to the corresponding author.
