# Supplementary material for: Two Versus One COVID-19 Vaccine Dose After Prior SARS-CoV-2 Infection: A Nationwide Retrospective Cohort Study
Source: Open Forum Infect Dis. 2026 Sep 22;13(10):ofag602. doi: 10.1093/ofid/ofag602 (PMC13634678; doi:10.1093/ofid/ofag602)
Supplement: Supplementary file 1 [file ofag602_supplementary_data.docx]

**Supplementary Appendix**

**Table of Contents**

[**Section S1. Study population and data sources**	2](#_Toc233888885)

[**Section S2. Laboratory methods and variant ascertainment**	5](#_Toc233888886)

[**Real-time reverse-transcription polymerase chain reaction testing**	5](#_Toc233888887)

[**Rapid antigen testing**	5](#_Toc233888888)

[**Classification of infections by variant type**	6](#_Toc233888889)

[**Section S3. Classification of coexisting conditions**	7](#_Toc233888890)

[**Table S1.** **Strengthening the Reporting of Observational Studies in Epidemiology (STROBE) checklist for cohort studies.**	9](#_Toc233888891)

[**Figure S1. Study population selection process comparing individuals with prior SARS-CoV-2 infection who received two vaccine doses with those who received a single dose.**	10](#_Toc233888892)

[**Table S2. Baseline characteristics of matched single-dose cohort participants according to subsequent receipt and timing of a documented second vaccine dose.**	11](#_Toc233888893)

[**Table S3. Subgroup analysis. Hazard ratios for SARS-CoV-2 reinfection and relative vaccine effectiveness comparing two versus one vaccine dose among individuals with prior SARS-CoV-2 infection, stratified by A) time between prior infection and vaccination, B) prior infection variant, and C) vaccine type.**	12](#_Toc233888894)

[**Table S4. Subgroup analysis. Hazard ratios for SARS-CoV-2 reinfection and relative vaccine effectiveness comparing individuals with prior SARS-CoV-2 infection who received two vaccine doses with those who received a single dose, stratified by A) age and B) clinical vulnerability status.**	13](#_Toc233888895)

[**Table S5. Interaction-model sensitivity analysis. Adjusted hazard ratios for SARS-CoV-2 reinfection and relative vaccine effectiveness comparing two versus one vaccine dose among individuals with prior SARS-CoV-2 infection, stratified by follow-up interval and selected subgroup variables, with P values for interaction.**	14](#_Toc233888896)

[**Table S6.** **Robust standard error sensitivity analysis. Hazard ratios and relative vaccine effectiveness for SARS-CoV-2 reinfection comparing two versus one vaccine dose among individuals with prior SARS-CoV-2 infection, using robust standard errors clustered at the individual level: A) in main analysis and B) stratified by time since vaccination.**	15](#_Toc233888897)

[**References**	16](#_Toc233888898)

# **Section S1. Study population and data sources**

Qatar’s national and universal public healthcare system uses the Cerner Millennium electronic medical record (EMR) system to track all the public healthcare encounters of each individual in the country, including all citizens and residents registered in the national and universal public healthcare system. Registration in the public healthcare system is mandatory for citizens and residents.

The databases analyzed in this study are data-extract downloads from the national EMR database that have been implemented on a regular weekly schedule since the onset of the pandemic by the Business Intelligence Unit at Hamad Medical Corporation (HMC). HMC is the national public healthcare provider in Qatar. At every download, all severe acute respiratory syndrome coronavirus 2 (SARS-CoV-2) tests, coronavirus disease 2019 (COVID-19) vaccinations, hospitalizations related to COVID-19, and all death records regardless of cause are provided to the authors through .csv files. These databases have been analyzed throughout the pandemic not only for study-related purposes, but also to provide policymakers with summary data and analytics to inform the national response.

Every health encounter in the national EMR is linked to an individual through the HMC Number, which serves as a unique identifier that links all records for this individual at the national level. Databases were merged and analyzed using the HMC Number to link all records pertaining to testing, vaccinations, hospitalizations, and deaths. All deaths in Qatar are recorded by the public healthcare system. COVID-19-related healthcare was provided exclusively in the public healthcare system. COVID-19 vaccination was also provided only through the public healthcare system. These health records were tracked throughout the COVID-19 pandemic using the national EMR system. This system was implemented in 2013, before the onset of the pandemic. This pre-established system ensured that we had access to comprehensive health records related to this study for both citizens and residents throughout the entire pandemic, allowing us to follow each person over time.

Demographic details for every HMC Number (individual) such as sex, age, and nationality are collected upon issuing of the universal health card, based on the Qatar Identity Card, which is a mandatory requirement by the Ministry of Interior to every citizen and resident in the country. Data extraction from the Qatar Identity Card to the digital health platform is performed electronically through scanning techniques.

SARS-CoV-2 testing in any facility in Qatar is tracked nationally in one database, the national testing database. This database covers all testing throughout the country, whether in public or private facilities. Every polymerase chain reaction (PCR) test conducted in Qatar, regardless of location or setting, is classified on the basis of symptoms and the reason for testing, such as the presence of clinical symptoms, contact tracing, participation in surveys or random testing campaigns, individual requests for testing, routine healthcare testing, pre-travel requirements, at the point of entry into the country, or any other relevant reasons for testing.

Before November 1, 2022, SARS-CoV-2 testing in Qatar was performed extensively with about 5% of the population tested every week ^1^. Based on the distribution of the reason for testing up to November 1, 2022, most of the tests in Qatar were conducted for routine reasons, such as travel-related purposes, and about 75% of infections were diagnosed not because of the presence of symptoms ^1,2^. Starting from November 1, 2022, testing for SARS-CoV-2 was substantially reduced with <1% of the population tested every week ^2^. This study factored all SARS-CoV-2-related testing included in the national testing database over the duration of the study.

December 19, 2021 marked the onset of the omicron wave in Qatar ^1^. The first omicron wave that reached its peak in January of 2022 was massive and strained the testing capacity in the country ^1,3-5^. To alleviate the burden on PCR testing, rapid antigen testing was rapidly introduced. The swift change in testing policy precluded incorporating reason for testing for a number of rapid antigen tests. While the reason for testing is documented for all PCR tests, it is not uniformly available for all rapid antigen tests. However, all medically supervised rapid antigen tests were captured by the national integrated digital health platform since January 5, 2022.

Rapid antigen test kits are accessible for purchase at pharmacies in Qatar, but results of home-based testing are neither reported nor documented in the national databases. Since SARS-CoV-2 test outcomes were linked to specific public health measures, restrictions, and privileges, testing policy and guidelines stress facility-based testing as the core testing mechanism in the population. While facility-based testing is provided free of charge or at low subsidized costs, depending on the reason for testing, home-based rapid antigen testing is de-emphasized and not supported as part of national policy.

Qatar launched its COVID-19 vaccination program in December 2020, employing mRNA vaccines and prioritizing individuals based on coexisting conditions and age criteria ^2,6^. COVID-19 vaccination was provided free of charge, regardless of citizenship or residency status, and was nationally tracked ^2,6^.

Qatar has unusually young, diverse demographics, in that only 9% of its residents are ≥50 years of age, and 89% are expatriates from over 150 countries ^2,7^. Further descriptions of the study population and these national databases were reported previously ^1,2,5,8-11^.

# **Section S2. Laboratory methods and variant ascertainment**

## **Real-time reverse-transcription polymerase chain reaction testing**

Nasopharyngeal and/or oropharyngeal swabs were collected for PCR testing and placed in Universal Transport Medium (UTM). Aliquots of UTM were: 1) extracted on KingFisher Flex (Thermo Fisher Scientific, USA), MGISP-960 (MGI, China), or ExiPrep 96 Lite (Bioneer, South Korea) followed by testing with real-time reverse-transcription PCR (RT-qPCR) using TaqPath COVID-19 Combo Kits (Thermo Fisher Scientific, USA) on an ABI 7500 FAST (Thermo Fisher Scientific, USA); 2) tested directly on the Cepheid GeneXpert system using the Xpert Xpress SARS-CoV-2 (Cepheid, USA); or 3) loaded directly into a Roche cobas 6800 system and assayed with the cobas SARS-CoV-2 Test (Roche, Switzerland). The first assay targets the viral S, N, and ORF1ab gene regions. The second targets the viral N and E-gene regions, and the third targets the ORF1ab and E-gene regions.

All PCR testing was conducted at the HMC Central Laboratory or Sidra Medicine Laboratory, following standardized protocols.

## **Rapid antigen testing**

SARS-CoV-2 antigen tests were performed on nasopharyngeal swabs using one of the following lateral flow antigen tests: Panbio COVID-19 Ag Rapid Test Device (Abbott, USA); SARS-CoV-2 Rapid Antigen Test (Roche, Switzerland); Standard Q COVID-19 Antigen Test (SD Biosensor, Korea); or CareStart COVID-19 Antigen Test (Access Bio, USA). All antigen tests were performed at point-of-care according to each manufacturer's instructions, at public or private hospitals and clinics throughout Qatar, with prior authorization and training by the Ministry of Public Health (MOPH). Antigen test results were electronically reported to the MOPH in real time using the Antigen Test Management System which is integrated with the national COVID-19 database.

## **Classification of infections by variant type**

Surveillance for SARS-CoV-2 variants in Qatar is based on viral genome sequencing and multiplex RT-qPCR variant screening ^12^ of weekly collected random positive clinical samples ^13-18^, complemented by deep sequencing of wastewater samples ^15,19,20^. Further details on the viral genome sequencing and multiplex RT-qPCR variant screening throughout the SARS-CoV-2 waves in Qatar can be found in previous publications ^1,4,13-18,21-27^.

# **Section S3. Classification of coexisting conditions**

The classification of patients and their coexisting conditions was carried out by an independent medical team appointed by the Ministry of Public Health. This process was part of routine needs assessment exercises aimed at planning healthcare use, utilization, and resource allocation.

As Qatar’s medical coding systems evolved over time, incorporating different versions of ICD codes as well as SNOMED codes, the medical team developed mappings to harmonize these codes and ensure consistent categorization of coexisting conditions. The study investigators did not have direct access to the detailed procedures, documentation, or specific codes and mappings used by this independent team. Instead, the finalized database of patients and their coexisting conditions was provided to the study investigators exclusively for research purposes.

Coexisting conditions were ascertained and classified based on the mapped codes for the conditions, as recorded in the electronic health record encounters of each individual in the national EMR database that includes all citizens and residents registered in the national and universal public healthcare system. The public healthcare system provides healthcare to the entire resident population of Qatar free of charge or at heavily subsidized costs, including prescription drugs. With the mass expansion of this sector in recent years, facilities have been built to cater to specific needs of subpopulations. For example, tens of facilities have been built, including clinics and hospitals, in localities with high density of craft and manual workers ^28^.

All encounters for each individual were analyzed to determine the coexisting-condition classification for that individual. The national EMR database includes encounters starting from 2013, when this system was launched in Qatar. Any individual who had at least one encounter with a specific coexisting-condition diagnosis since 2013 was classified as having that coexisting condition. Individuals who do not have records of coexisting-condition encounters in the public healthcare system were classified as having no coexisting conditions.

The classification of coexisting conditions spanned the following conditions: 1) Behçet’s disease, 2) cancer, 3) cardiovascular diseases, 4) infectious and parasitic diseases, 5) Crohn’s disease, 6) chronic kidney disease (CKD), 7) chronic liver disease (CLD), 8) chronic lung disease, 9) congenital malformations, deformations and chromosomal abnormalities, 10) diseases of the blood and blood-forming organs and certain disorders involving the immune mechanism, 11) diseases of the ear and mastoid process, 12) deep vein thrombosis (DVT), 13) dermatitis, 14) diabetes mellitus, 15) diseases of the circulatory system, 16) diseases of the digestive system, 17) diseases of the eye and adnex, 18) diseases of the genitourinary system, 19) diseases of the musculoskeletal system and connective tissue, 20) diseases of the nervous system, 21) diseases of the respiratory system, 22) diseases of the skin and subcutaneous tissue, 23) endocrine, nutritional and metabolic diseases, 24) gingivitis, 25) hypertension, 26) injury, poisoning and certain other consequences of external causes, 27) mental and behavioral disorders, 28) neoplasms, 29) periodontitis, 30) pregnancy, childbirth and the puerperium, 31) pulmonary tuberculosis, 32) rheumatoid arthritis, 33) Sjogren’s syndrome, 34) stroke or neural conditions, 35) symptoms, signs and abnormal clinical and laboratory findings, not elsewhere classified, 36) systemic lupus erythematosus, 37) systemic sclerosis, 38) organ transplant, and 39) other unspecified factors influencing health status and contact with health services.

# **Table S1.** **Strengthening the Reporting of Observational Studies in Epidemiology (STROBE) checklist for cohort studies.**

|  | Item | Recommendation | Main Text page |
| --- | --- | --- | --- |
| **Title and abstract** | 1 | (*a*) Indicate the study’s design with a commonly used term in the title or the abstract (*b*) Provide in the abstract an informative and balanced summary of what was done and what was found | Abstract |
| Introduction | | | |
| Background/rationale | 2 | Explain the scientific background and rationale for the investigation being reported | Introduction |
| Objectives | 3 | State specific objectives, including any prespecified hypotheses | Introduction |
| Methods | | | |
| Study design | 4 | Present key elements of study design early in the paper | Methods (‘Study population and data sources’ & ‘Study design’) |
| Setting | 5 | Describe the setting, locations, and relevant dates, including periods of recruitment, exposure, follow-up, and data collection | Methods (‘Study population and data sources’ & ‘Study design’) & Figure S1 in Supplementary Appendix |
| Participants | 6 | (*a*) Give the eligibility criteria, and the sources and methods of selection of participants. Describe methods of follow-up (*b*) For matched studies, give matching criteria and number of exposed and unexposed | Methods (‘Cohorts’ eligibility and matching’ & ‘Cohorts’ follow-up’), & Section S1 & Figure S1 in Supplementary Appendix |
| Variables | 7 | Clearly define all outcomes, exposures, predictors, potential confounders, and effect modifiers. Give diagnostic criteria, if applicable | Methods (‘Study design’, ‘Cohorts’ eligibility and matching’, ‘Cohorts’ follow-up’, & ‘Statistical analysis’), Table 1, & Sections S1-S3 in Supplementary Appendix |
| Data sources/ measurement | 8* | For each variable of interest, give sources of data and details of methods of assessment (measurement). Describe comparability of assessment methods if there is more than one group | Methods (‘Study population and data sources’, ‘Study design’, & ‘Statistical analysis’), Table 1, & Sections S1-S3 in Supplementary Appendix |
| Bias | 9 | Describe any efforts to address potential sources of bias | Methods (‘Cohorts’ eligibility and matching’, ‘Cohorts’ follow-up’, & ‘Statistical analysis’) |
| Study size | 10 | Explain how the study size was arrived at | Figure S1 in Supplementary Appendix |
| Quantitative variables | 11 | Explain how quantitative variables were handled in the analyses. If applicable, describe which groupings were chosen and why | Methods (‘Cohorts’ eligibility and matching’ & ‘Statistical analysis’) & Table 1 |
| Statistical methods | 12 | (*a*) Describe all statistical methods, including those used to control for confounding (*b*) Describe methods used to examine subgroups and interactions | Methods (‘Statistical analysis’) |
|  |  | (*c*) Explain how missing data were addressed (*d*) If applicable, explain how loss to follow-up was addressed | Not applicable, see Methods (‘Study population, and data sources’) & Section S1 in Supplementary Appendix |
|  |  | (*e*) Describe any sensitivity analyses | Methods (‘Statistical analysis’) |
| Results | | |  |
| Participants | 13* | (a) Report numbers of individuals at each stage of study—eg numbers potentially eligible, examined for eligibility, confirmed eligible, included in the study, completing follow-up, and analysed (b) Give reasons for non-participation at each stage (c) Consider use of a flow diagram | Results (‘Study population’), Table 1, & Figure S1 in Supplementary Appendix |
| Descriptive data | 14 | (a) Give characteristics of study participants (eg demographic, clinical, social) and information on exposures and potential confounders | Table 1 and Table S2 in Supplementary Appendix |
|  |  | (b) Indicate number of participants with missing data for each variable of interest | Not applicable, see Methods (‘Study population and data sources’) & Section S1 in Supplementary Appendix |
|  |  | (c) Summarise follow-up time (eg, average and total amount) | Results (‘Incidence of SARS-CoV-2 reinfection’), Figure 1, & Table 2 |
| Outcome data | 15 | Report numbers of outcome events or summary measures over time | Results (‘Incidence of SARS-CoV-2 reinfection’) & Table 2 |
| Main results | 16 | (a) Give unadjusted estimates and, if applicable, confounder-adjusted estimates and their precision (eg, 95% confidence interval). Make clear which confounders were adjusted for and why they were included | Results (‘Incidence of SARS-CoV-2 reinfection’) & Table 2 |
|  |  | (b) Report category boundaries when continuous variables were categorized | Table 1 |
|  |  | (c) If relevant, consider translating estimates of relative risk into absolute risk for a meaningful time period | Not applicable |
| Other analyses | 17 | Report other analyses done—eg analyses of subgroups and interactions, and sensitivity analyses | Results (‘Incidence of SARS-CoV-2 reinfection’ & ‘Subgroup and sensitivity analyses’), Figures 2-3, & Tables S3-S6 in Supplementary Appendix |
| **Discussion** | | | |
| Key results | 18 | Summarise key results with reference to study objectives | Discussion, paragraphs 1-5 |
| Limitations | 19 | Discuss limitations of the study, taking into account sources of potential bias or imprecision. Discuss both direction and magnitude of potential bias | Discussion, paragraphs 6-10 |
| Interpretation | 20 | Give a cautious overall interpretation of results considering objectives, limitations, multiplicity of analyses, results from similar studies, and other relevant evidence | Conclusion |
| Generalisability | 21 | Discuss the generalisability (external validity) of the study results | Discussion, paragraphs 6-9 |
| **Other information** | | | |
| Funding | 22 | Give the source of funding and role of the funders for the present study | Funding statement |

# **Figure S1. Study population selection process comparing individuals with prior SARS-CoV-2 infection who received two vaccine doses with those who received a single dose.**

Abbreviations: COVID-19, coronavirus disease; SARS-CoV-2, severe acute respiratory syndrome coronavirus 2.

^*^Persons in the two-dose cohort were matched exactly one-to-one to persons in the single-dose cohort by sex, 10-year age group, nationality, number of coexisting conditions, prior infection variant, time between infection and vaccination, vaccine type, and calendar week of vaccination (calendar week of the second dose in the two-dose cohort matched to the calendar week of the first dose in the single-dose cohort). Each matched pair was followed from the date of second dose vaccination for the individual in the two-dose cohort.

# **Table S2. Baseline characteristics of matched single-dose cohort participants according to subsequent receipt and timing of a documented second vaccine dose.**

| **Characteristics** | **No documented second dose during study period** | **Documented second dose within 30 days of the first dose** | **Documented second dose 31 or more days after the first dose** |
| --- | --- | --- | --- |
|  | **N=871** | **N=54,306** | **N=6,677** |
| Median age (IQR) — years | 36 (31-41) | 37 (31-44) | 36 (32-43) |
| Age group — no. (%) |  |  |  |
| 0-19 years | 52 (6.0) | 2,648 (4.9) | 225 (3.4) |
| 20-29 years | 114 (13.1) | 7,643 (14.1) | 873 (13.1) |
| 30-39 years | 432 (49.6) | 21,479 (39.6) | 3,226 (48.3) |
| 40-49 years | 201 (23.1) | 15,049 (27.7) | 1,722 (25.8) |
| 50-59 years | 62 (7.1) | 5,735 (10.6) | 531 (8.0) |
| 60-69 years | 7 (0.8) | 1,504 (2.8) | 95 (1.4) |
| 70+ years | 3 (0.3) | 248 (0.5) | 5 (0.1) |
| Sex |  |  |  |
| Male | 490 (56.3) | 41,656 (76.7) | 5,058 (75.8) |
| Female | 381 (43.7) | 12,650 (23.3) | 1,619 (24.2) |
| Nationality^*^ |  |  |  |
| Bangladeshi | 65 (7.5) | 6,451 (11.9) | 858 (12.9) |
| Egyptian | 32 (3.7) | 2,418 (4.5) | 234 (3.5) |
| Filipino | 210 (24.1) | 5,420 (10.0) | 835 (12.5) |
| Indian | 346 (39.7) | 15,954 (29.4) | 2,091 (31.3) |
| Nepalese | 47 (5.4) | 7,001 (12.9) | 1,020 (15.3) |
| Pakistani | 31 (3.6) | 2,700 (5.0) | 389 (5.8) |
| Qatari | 79 (9.1) | 7,651 (14.1) | 446 (6.7) |
| Sri Lankan | 9 (1.0) | 1,781 (3.3) | 201 (3.0) |
| Sudanese | 14 (1.6) | 1,231 (2.3) | 139 (2.1) |
| Other nationalities^†^ | 38 (4.4) | 3,699 (6.8) | 464 (6.9) |
| Coexisting conditions |  |  |  |
| 0 | 815 (93.6) | 46,030 (84.8) | 6,001 (89.9) |
| 1 | 37 (4.2) | 4,964 (9.1) | 437 (6.5) |
| 2 | 11 (1.3) | 1,956 (3.6) | 158 (2.4) |
| 3 | 3 (0.3) | 646 (1.2) | 44 (0.7) |
| 4 | 0 (0.0) | 336 (0.6) | 18 (0.3) |
| 5 | 2 (0.2) | 168 (0.3) | 8 (0.1) |
| ≥6 | 3 (0.3) | 206 (0.4) | 11 (0.2) |
| Vaccine type |  |  |  |
| BNT162b2 | 435 (49.9) | 37,534 (69.1) | 1,576 (23.6) |
| mRNA-1273 | 411 (47.2) | 16,697 (30.7) | 4,795 (71.8) |
| AZD1222 | 21 (2.4) | 70 (0.1) | 305 (4.6) |
| Other vaccine types^‡^ | 4 (0.5) | 5 (0.009) | 1 (0.01) |
| Prior infection variant |  |  |  |
| Pre-omicron | 604 (69.3) | 53,986 (99.4) | 6,602 (98.9) |
| Omicron | 267 (30.7) | 320 (0.6) | 75 (1.1) |
| Time between prior infection and vaccination^§^ |  |  |  |
| <6 months | 286 (32.8) | 17,559 (32.3) | 2,264 (33.9) |
| 6-11 months | 381 (43.7) | 25,770 (47.5) | 2,496 (37.4) |
| ≥12 months | 204 (23.4) | 10,977 (20.2) | 1,917 (28.7) |

Abbreviations: IQR, interquartile range; SARS-CoV-2, severe acute respiratory syndrome coronavirus 2.

^*^Nationalities were chosen to represent the most populous groups in Qatar.

^†^These comprise up to 50 other nationalities.

^‡^These include mRNA-1273.214 (Spikevax bivalent original/omicron BA.1), mRNA-1273.222 (Spikevax bivalent original/omicron BA.4/BA.5), BNT162b2 original/omicron BA.4/BA.5 (Comirnaty original/omicron BA.4/BA.5), and mRNA-1273.815 (Spikevax XBB.1.5).

^§^Vaccination refers to the first dose in the matched single-dose cohort.

# **Table S3. Subgroup analysis. Hazard ratios for SARS-CoV-2 reinfection and relative vaccine effectiveness comparing two versus one vaccine dose among individuals with prior SARS-CoV-2 infection, stratified by A) time between prior infection and vaccination, B) prior infection variant, and C) vaccine type.**

| **Epidemiological measure** | **Two-dose cohort^*^** | | **Single-dose cohort^*^** |
| --- | --- | --- | --- |
| A) **Stratified by time between prior infection and vaccination**^†^ |  | |  |
| **<6 months** |  | |  |
| Sample size | 20,109 | | 20,109 |
| Total follow-up time (person-weeks) | 106,432 | | 105,276 |
| Incident infections | 36 | | 52 |
| Incidence rate of reinfection (per 10,000 person-weeks) | 3.4 (2.4 to 4.7) | | 4.9 (3.8 to 6.5) |
| Unadjusted hazard ratio for SARS-CoV-2 reinfection (95% CI) | 0.68 (0.44 to 1.04) | | |
| Adjusted hazard ratio for SARS-CoV-2 reinfection (95% CI)^‡^ | 0.64 (0.42 to 0.99) | | |
| Relative vaccine effectiveness against SARS-CoV-2 reinfection in % (95% CI) | 35.6 (0.6 to 58.3) | | |
| **≥6 months** |  | |  |
| Sample size | 41,745 | | 41,745 |
| Total follow-up time (person-weeks) | 219,679 | | 216,866 |
| Incident infections | 57 | | 87 |
| Incidence rate of reinfection (per 10,000 person-weeks) | 2.6 (2.0 to 3.4) | | 4.0 (3.2 to 4.9) |
| Unadjusted hazard ratio for SARS-CoV-2 reinfection (95% CI) | 0.65 (0.46 to 0.90) | | |
| Adjusted hazard ratio for SARS-CoV-2 reinfection (95% CI)^‡^ | 0.59 (0.42 to 0.82) | | |
| Relative vaccine effectiveness against SARS-CoV-2 reinfection in % (95% CI) | 41.5 (17.9 to 58.2) | | |
| B) **Stratified by prior infection variant** |  | |  |
| **Prior pre-omicron infection** |  | |  |
| Sample size | 61,192 | | 61,192 |
| Total follow-up time (person-weeks) | 291,580 | | 288,532 |
| Incident infections | 84 | | 119 |
| Incidence rate of reinfection (per 10,000 person-weeks) | 2.9 (2.3 to 3.6) | | 4.1 (3.4 to 4.9) |
| Unadjusted hazard ratio for SARS-CoV-2 reinfection (95% CI) | 0.69 (0.53 to 0.92) | | |
| Adjusted hazard ratio for SARS-CoV-2 reinfection (95% CI)^‡^ | 0.64 (0.48 to 0.85) | | |
| Relative vaccine effectiveness against SARS-CoV-2 reinfection in % (95% CI) | 36.1 (15.3 to 51.8) | | |
| **Prior omicron infection** |  | |  |
| Sample size | 662 | | 662 |
| Total follow-up time (person-weeks) | 34,531 | | 33,611 |
| Incident infections | 9 | | 20 |
| Incidence rate of reinfection (per 10,000 person-weeks) | 2.6 (1.4 to 5.0) | | 5.9 (3.8 to 9.2) |
| Unadjusted hazard ratio for SARS-CoV-2 reinfection (95% CI) | 0.44 (0.20 to 0.97) | | |
| Adjusted hazard ratio for SARS-CoV-2 reinfection (95% CI)^‡^ | 0.43 (0.19 to 0.96) | | |
| Relative vaccine effectiveness against SARS-CoV-2 reinfection in % (95% CI) | 57.5 (3.7 to 81.2) | | |
| C) **Stratified by vaccine type** |  | | |
| **BNT162b2** |  |  | |
| Sample size | 39,545 | 39,545 | |
| Total follow-up time (person-weeks) | 178,763 | 175,524 | |
| Incident infections | 73 | 103 | |
| Incidence rate of reinfection (per 10,000 person-weeks) | 4.1 (3.2 to 5.1) | 5.9 (4.8 to 7.1) | |
| Unadjusted hazard ratio for SARS-CoV-2 reinfection (95% CI) | 0.69 (0.51 to 0.93) | | |
| Adjusted hazard ratio for SARS-CoV-2 reinfection (95% CI)^‡^ | 0.63 (0.46 to 0.85) | | |
| Relative vaccine effectiveness against SARS-CoV-2 reinfection in % (95% CI) | 37.2 (14.8 to 53.7) | | |
| **mRNA-1273** |  | | |
| Sample size | 21,903 | 21,903 | |
| Total follow-up time (person-weeks) | 141,027 | 140,182 | |
| Incident infections | 18 | 33 | |
| Incidence rate of reinfection (per 10,000 person-weeks) | 1.3 (0.8 to 2.0) | 2.4 (1.7 to 3.3) | |
| Unadjusted hazard ratio for SARS-CoV-2 reinfection (95% CI) | 0.54 (0.31 to 0.96) | | |
| Adjusted hazard ratio for SARS-CoV-2 reinfection (95% CI)^‡^ | 0.50 (0.28 to 0.89) | | |
| Relative vaccine effectiveness against SARS-CoV-2 reinfection in % (95% CI) | 50.3 (10.7 to 72.3) | | |

Abbreviations: CI, confidence interval; SARS-CoV-2, severe acute respiratory syndrome coronavirus 2.

^*^Persons in the two-dose cohort were matched exactly one-to-one to persons in the single-dose cohort by sex, 10-year age group, nationality, number of coexisting conditions, prior infection variant, time between prior infection and vaccination, vaccine type, and calendar week of vaccination (calendar week of the second dose in the two-dose cohort matched to the calendar week of the first dose in the single-dose cohort). Each matched pair was followed from the date of second dose vaccination for the individual in the two-dose cohort.

^†^Vaccination refers to the second dose in the two-dose cohort and the first dose in the single-dose cohort.

^‡^Adjusted for sex, 10-year age group, nationality, number of coexisting conditions, prior infection variant, time between prior infection and vaccination, vaccine type, and the calendar week of vaccination (calendar week of the second dose in the two-dose cohort matched to the calendar week of the first dose in the single-dose cohort), and testing rate, as applicable depending on strata.

# **Table S4. Subgroup analysis. Hazard ratios for SARS-CoV-2 reinfection and relative vaccine effectiveness comparing individuals with prior SARS-CoV-2 infection who received two vaccine doses with those who received a single dose, stratified by A) age and B) clinical vulnerability status.**

| **Epidemiological measure** | **Two-dose cohort^*^** | | **Single-dose cohort^*^** |
| --- | --- | --- | --- |
| A) **Stratified by age** |  | |  |
| **Less than 50 years** |  | |  |
| Sample size | 53,664 | | 53,664 |
| Total follow-up time (person-weeks) | 291,026 | | 286,933 |
| Incident infections | 86 | | 126 |
| Incidence rate of reinfection (per 10,000 person-weeks) | 3.0 (2.4 to 3.7) | | 4.4 (3.7 to 5.2) |
| Unadjusted hazard ratio for SARS-CoV-2 reinfection (95% CI) | 0.67 (0.51 to 0.88) | | |
| Adjusted hazard ratio for SARS-CoV-2 reinfection (95% CI)^†^ | 0.62 (0.47 to 0.81) | | |
| Relative vaccine effectiveness against SARS-CoV-2 reinfection in % (95% CI) | 38.4 (18.8 to 53.4) | | |
| **50 or more years** |  | |  |
| Sample size | 8,190 | | 8,190 |
| Total follow-up time (person-weeks) | 35,086 | | 35,209 |
| Incident infections | 7 | | 13 |
| Incidence rate of reinfection (per 10,000 person-weeks) | 2.0 (1.0 to 4.2) | | 3.7 (2.1 to 6.4) |
| Unadjusted hazard ratio for SARS-CoV-2 reinfection (95% CI) | 0.54 (0.21 to 1.35) | | |
| Adjusted hazard ratio for SARS-CoV-2 reinfection (95% CI)^†^ | 0.33 (0.11 to 0.98) | | |
| Relative vaccine effectiveness against SARS-CoV-2 reinfection in % (95% CI) | 65.8 (1.1 to 88.2) | | |
| B) **Stratified by clinical vulnerability status** |  | | |
| **Less clinically vulnerable to severe COVID-19** |  |  | |
| Sample size | 52,054 | 52,054 | |
| Total follow-up time (person-weeks) | 284,987 | 281,200 | |
| Incident infections | 84 | 122 | |
| Incidence rate of reinfection (per 10,000 person-weeks) | 2.9 (2.4 to 3.7) | 4.3 (3.6 to 5.2) | |
| Unadjusted hazard ratio for SARS-CoV-2 reinfection (95% CI) | 0.68 (0.51 to 0.90) | | |
| Adjusted hazard ratio for SARS-CoV-2 reinfection (95% CI)^†^ | 0.63 (0.47 to 0.83) | | |
| Relative vaccine effectiveness against SARS-CoV-2 reinfection in % (95% CI) | 37.4 (17.1 to 52.7) | | |
| **More clinically vulnerable to severe COVID-19** |  | | |
| Sample size | 9,800 | 9,800 | |
| Total follow-up time (person-weeks) | 41,124 | 40,942 | |
| Incident infections | 9 | 17 | |
| Incidence rate of reinfection (per 10,000 person-weeks) | 2.2 (1.1 to 4.2) | 4.2 (2.6 to 6.7) | |
| Unadjusted hazard ratio for SARS-CoV-2 reinfection (95% CI) | 0.52 (0.23 to 1.17) | | |
| Adjusted hazard ratio for SARS-CoV-2 reinfection (95% CI)^†^ | 0.33 (0.14 to 0.83) | | |
| Relative vaccine effectiveness against SARS-CoV-2 reinfection in % (95% CI) | 66.3 (16.9 to 86.4) | | |

Abbreviations: CI, confidence interval; COVID-19, coronavirus disease; SARS-CoV-2, severe acute respiratory syndrome coronavirus 2.

^*^Persons in the two-dose cohort were matched exactly one-to-one to persons in the single-dose cohort by sex, 10-year age group, nationality, number of coexisting conditions, prior infection variant, time between prior infection and vaccination, vaccine type, and calendar week of vaccination (calendar week of the second dose in the two-dose cohort matched to the calendar week of the first dose in the single-dose cohort). Each matched pair was followed from the date of second dose vaccination for the individual in the two-dose cohort.

^†^Adjusted for sex, 10-year age group, nationality, number of coexisting conditions, prior infection variant, time between prior infection and vaccination, vaccine type, and the calendar week of vaccination (calendar week of the second dose in the two-dose cohort matched to the calendar week of the first dose in the single-dose cohort), and testing rate, as applicable depending on strata.

# **Table S5. Interaction-model sensitivity analysis. Adjusted hazard ratios for SARS-CoV-2 reinfection and relative vaccine effectiveness comparing two versus one vaccine dose among individuals with prior SARS-CoV-2 infection, stratified by follow-up interval and selected subgroup variables, with P values for interaction.**

| **Analysis stratum** | **aHR**  **(95% CI)^*^** | **rVE in %**  **(95% CI)** | **P for interaction^†^** |
| --- | --- | --- | --- |
| **Time since vaccination** |  |  |  |
| <24 days | 0.75 (0.48 to 1.18) | 24.8 (-15.0 to 52.0) | 0.417 |
| 24-179 days | 0.48 (0.29 to 0.79) | 52.3 (20.9 to 71.3) |  |
| ≥180 days | 0.61 (0.39 to 0.94) | 39.3 (6.4 to 60.7) |  |
| **Time between prior infection and vaccination** |  |  |  |
| <6 months | 0.66 (0.43 to 1.01) | 34.0 (-1.2 to 56.9) | 0.640 |
| ≥6 months | 0.58 (0.41 to 0.81) | 42.0 (18.7 to 58.6) |  |
| **Prior infection variant** |  |  |  |
| Prior pre-omicron infection | 0.64 (0.48 to 0.85) | 36.2 (15.3 to 51.9) | 0.342 |
| Prior omicron infection | 0.42 (0.19 to 0.94) | 57.6 (6.0 to 80.9) |  |
| **Vaccine type** |  |  |  |
| BNT162b2 | 0.63 (0.47 to 0.86) | 36.8 (14.4 to 53.4) | 0.647 |
| mRNA-1273 | 0.54 (0.31 to 0.96) | 45.7 (3.5 to 69.5) |  |
| **Age** |  |  |  |
| <50 years | 0.62 (0.47 to 0.82) | 38.2 (18.5 to 53.2) | 0.747 |
| 50 or more years | 0.53 (0.21 to 1.33) | 47.3 (-24.6 to 79.0) |  |
| **Clinical vulnerability status** |  |  |  |
| Less clinically vulnerable to severe COVID-19 | 0.63 (0.47 to 0.83) | 37.3 (17.0 to 52.7) | 0.525 |
| More clinically vulnerable to severe COVID-19 | 0.47 (0.21 to 1.07) | 52.6 (-6.6 to 79.0) |  |

Abbreviations: aHR, adjusted hazard ratio; CI, confidence interval; rVE, relative vaccine effectiveness; SARS-CoV-2, severe acute respiratory syndrome coronavirus 2.

^*^Adjusted hazard ratios were derived from Cox regression models including interaction terms between vaccination cohort and follow-up interval or subgroup variable, as applicable.

^†^P values for interaction were derived from Wald tests of the interaction terms.

# **Table S6.** **Robust standard error sensitivity analysis. Hazard ratios and relative vaccine effectiveness for SARS-CoV-2 reinfection comparing two versus one vaccine dose among individuals with prior SARS-CoV-2 infection, using robust standard errors clustered at the individual level: A) in main analysis and B) stratified by time since vaccination.**

| **Epidemiological measure** | **Two-dose cohort^*^** | **Single-dose cohort^*^** |
| --- | --- | --- |
| A) **Main analysis** |  |  |
| Sample size | 61,854 | 61,854 |
| Total follow-up time (person-weeks) | 326,111 | 322,142 |
| Incident infections | 93 | 139 |
| Incidence rate of reinfection (per 10,000 person-weeks; 95% CI) | 2.9 (2.3 to 3.5) | 4.3 (3.7 to 5.1) |
| Unadjusted hazard ratio for SARS-CoV-2 reinfection (95% CI) | 0.66 (0.51 to 0.86) | |
| Adjusted hazard ratio for SARS-CoV-2 reinfection (95% CI)^†^ | 0.61 (0.46 to 0.80) | |
| Relative vaccine effectiveness against SARS-CoV-2 reinfection in % (95% CI) | 38.9 (19.6 to 53.6) | |
| B) **Time since vaccination**^‡^ |  | |
| <24 days |  | |
| Adjusted hazard ratio for SARS-CoV-2 reinfection (95% CI)^†^ | 0.73 (0.47 to 1.15) | |
| Relative vaccine effectiveness against SARS-CoV-2 reinfection in % (95% CI) | 26.9 (-12.8 to 53.5) | |
| 24-179 days |  | |
| Adjusted hazard ratio for SARS-CoV-2 reinfection (95% CI)^†^ | 0.48 (0.28 to 0.83) | |
| Relative vaccine effectiveness against SARS-CoV-2 reinfection in % (95% CI) | 51.8 (16.7 to 72.1) | |
| ≥180 days |  | |
| Adjusted hazard ratio for SARS-CoV-2 reinfection (95% CI)^†^ | 0.63 (0.39 to 1.03) | |
| Relative vaccine effectiveness against SARS-CoV-2 reinfection in % (95% CI) | 36.8 (-2.8 to 61.2) | |

Abbreviations: CI, confidence interval; SARS-CoV-2, severe acute respiratory syndrome coronavirus 2.

^*^Persons in the two-dose cohort were matched exactly one-to-one to persons in the single-dose cohort by sex, 10-year age group, nationality, number of coexisting conditions, prior infection variant, time between infection and vaccination, vaccine type, and calendar week of vaccination (calendar week of the second dose in the two-dose cohort matched to the calendar week of the first dose in the single-dose cohort). Each matched pair was followed from the date of second dose vaccination for the individual in the two-dose cohort.

^†^Adjusted for sex, 10-year age group, nationality, number of coexisting conditions, prior infection variant, time between prior infection and vaccination, vaccine type, and the calendar week of vaccination (calendar week of the second dose in the two-dose cohort matched to the calendar week of the first dose in the single-dose cohort), and testing rate, as applicable depending on strata.

^‡^Vaccination refers to the second dose in the two-dose cohort and the first dose in the single-dose cohort.

# **References**

1.	Altarawneh HN, Chemaitelly H, Ayoub HH, et al. Effects of previous infection and vaccination on symptomatic Omicron infections. *The New England journal of medicine.* Jul 7 2022;387(1):21-34.

2.	Abu-Raddad LJ, Chemaitelly H, Ayoub HH, et al. Characterizing the Qatar advanced-phase SARS-CoV-2 epidemic. *Scientific reports.* Mar 18 2021;11(1):6233.

3.	Chemaitelly H, Faust JS, Krumholz HM, et al. Short- and longer-term all-cause mortality among SARS-CoV-2- infected individuals and the pull-forward phenomenon in Qatar: a national cohort study. *International journal of infectious diseases : IJID : official publication of the International Society for Infectious Diseases.* Nov 2023;136:81-90.

4.	Altarawneh HN, Chemaitelly H, Hasan MR, et al. Protection against the Omicron variant from previous SARS-CoV-2 infection. *The New England journal of medicine.* Mar 31 2022;386(13):1288-1290.

5.	Mahmoud MA, Ayoub HH, Coyle P, et al. SARS-CoV-2 infection and effects of age, sex, comorbidity, and vaccination among older individuals: A national cohort study. *Influenza and other respiratory viruses.* Nov 2023;17(11):e13224.

6.	Abu-Raddad LJ, Chemaitelly H, Bertollini R, National Study Group for Covid Vaccination. Effectiveness of mRNA-1273 and BNT162b2 Vaccines in Qatar. *N Engl J Med.* Feb 24 2022;386(8):799-800.

7.	Planning and Statistics Authority-State of Qatar. Qatar Monthly Statistics. Available from: <https://www.psa.gov.qa/en/pages/default.aspx>. Accessed on: May 26, 2020. 2020.

8.	Chemaitelly H, Bertollini R, Abu-Raddad LJ, National Study Group for Covid Epidemiology. Efficacy of Natural Immunity against SARS-CoV-2 Reinfection with the Beta Variant. *N Engl J Med.* Dec 30 2021;385(27):2585-2586.

9.	Chemaitelly H, Ayoub HH, AlMukdad S, et al. Protection from previous natural infection compared with mRNA vaccination against SARS-CoV-2 infection and severe COVID-19 in Qatar: a retrospective cohort study. *The Lancet. Microbe.* Dec 2022;3(12):e944-e955.

10.	Chemaitelly H, Ayoub HH, Tang P, et al. History of primary-series and booster vaccination and protection against Omicron reinfection. *Science advances.* Oct 6 2023;9(40):eadh0761.

11.	AlNuaimi AA, Chemaitelly H, Semaan S, et al. All-cause and COVID-19 mortality in Qatar during the COVID-19 pandemic. *BMJ Glob Health.* May 2023;8(5).

12.	Vogels C, Fauver J, Grubaugh N. Multiplexed RT-qPCR to screen for SARS-COV-2 B.1.1.7, B.1.351, and P.1 variants of concern V.3. dx.doi.org/10.17504/protocols.io.br9vm966. 2021(June 6, 2021).

13.	Abu-Raddad LJ, Chemaitelly H, Butt AA, National Study Group for Covid Vaccination. Effectiveness of the BNT162b2 Covid-19 Vaccine against the B.1.1.7 and B.1.351 Variants. *N Engl J Med.* Jul 8 2021;385(2):187-189.

14.	Chemaitelly H, Yassine HM, Benslimane FM, et al. mRNA-1273 COVID-19 vaccine effectiveness against the B.1.1.7 and B.1.351 variants and severe COVID-19 disease in Qatar. *Nature medicine.* Sep 2021;27(9):1614-1621.

15.	National Project of Surveillance for Variants of Concern and Viral Genome Sequencing. Qatar viral genome sequencing data. Data on randomly collected samples. <https://www.gisaid.org/phylodynamics/global/nextstrain/>. 2021; <https://www.gisaid.org/phylodynamics/global/nextstrain/>.

16.	Benslimane FM, Al Khatib HA, Al-Jamal O, et al. One year of SARS-CoV-2: genomic characterization of COVID-19 outbreak in Qatar. *Frontiers in cellular and infection microbiology.* 2021;11:768883.

17.	Hasan MR, Kalikiri MKR, Mirza F, et al. Real-Time SARS-CoV-2 Genotyping by High-Throughput Multiplex PCR Reveals the Epidemiology of the Variants of Concern in Qatar. *Int J Infect Dis.* Nov 2021;112:52-54.

18.	Chemaitelly H, Tang P, Hasan MR, et al. Waning of BNT162b2 vaccine protection against SARS-CoV-2 infection in Qatar. *The New England journal of medicine.* Dec 9 2021;385(24):e83.

19.	Saththasivam J, El-Malah SS, Gomez TA, et al. COVID-19 (SARS-CoV-2) outbreak monitoring using wastewater-based epidemiology in Qatar. *Sci Total Environ.* Jun 20 2021;774:145608.

20.	El-Malah SS, Saththasivam J, Jabbar KA, et al. Application of human RNase P normalization for the realistic estimation of SARS-CoV-2 viral load in wastewater: A perspective from Qatar wastewater surveillance. *Environ Technol Innov.* Aug 2022;27:102775.

21.	Abu-Raddad LJ, Chemaitelly H, Ayoub HH, et al. Effect of mRNA vaccine boosters against SARS-CoV-2 Omicron infection in Qatar. *The New England journal of medicine.* May 12 2022;386(19):1804-1816.

22.	Tang P, Hasan MR, Chemaitelly H, et al. BNT162b2 and mRNA-1273 COVID-19 vaccine effectiveness against the SARS-CoV-2 Delta variant in Qatar. *Nature medicine.* Dec 2021;27(12):2136-2143.

23.	Chemaitelly H, Ayoub HH, AlMukdad S, et al. Duration of mRNA vaccine protection against SARS-CoV-2 Omicron BA.1 and BA.2 subvariants in Qatar. *Nature communications.* Jun 2 2022;13(1):3082.

24.	Qassim SH, Chemaitelly H, Ayoub HH, et al. Effects of BA.1/BA.2 subvariant, vaccination and prior infection on infectiousness of SARS-CoV-2 omicron infections. *J Travel Med.* Sep 17 2022;29(6).

25.	Altarawneh HN, Chemaitelly H, Ayoub HH, et al. Protective effect of previous SARS-CoV-2 infection against Omicron BA.4 and BA.5 subvariants. *The New England journal of medicine.* Oct 27 2022;387(17):1620-1622.

26.	Chemaitelly H, Tang P, Coyle P, et al. Protection against Reinfection with the Omicron BA.2.75 Subvariant. *N Engl J Med.* Feb 16 2023;388(7):665-667.

27.	Chemaitelly H, Coyle P, Ben Kacem MA, et al. Protection of natural infection against reinfection with SARS-CoV-2 JN.1 variant. *J Travel Med.* Jun 3 2024;31(4).

28.	Al-Thani MH, Farag E, Bertollini R, et al. SARS-CoV-2 Infection Is at Herd Immunity in the Majority Segment of the Population of Qatar. *Open Forum Infect Dis.* Aug 2021;8(8):ofab221.
